# Is atypical rhythm a risk factor for developmental speech and language disorders?

**DOI:** 10.1002/wcs.1528

**Published:** 2020-04-03

**Authors:** Enikȍ Ladányi, Valentina Persici, Anna Fiveash, Barbara Tillmann, Reyna L. Gordon

**Affiliations:** 1 Department of Otolaryngology, Vanderbilt University Medical Center, Nashville, Tennessee; 2 Department of Psychology, Università degli Studi di Milano – Bicocca, Milan, Italy; 3 Vanderbilt Brain Institute, Vanderbilt University, Nashville, Tennessee; 4 Lyon Neuroscience Research Center, Auditory Cognition and Psychoacoustics Team, CRNL, INSERM, University of Lyon 1, U1028, CNRS, UMR5292, Lyon, France; 5 Vanderbilt Genetics Institute, Vanderbilt University, Nashville, Tennessee; 6 Vanderbilt Kennedy Center, Vanderbilt University Medical Center, Nashville, Tennessee

**Keywords:** developmental language disorder, dyslexia, rhythm, risk factor, specific language impairment

## Abstract

Although a growing literature points to substantial variation in speech/language abilities related to individual differences in musical abilities, mainstream models of communication sciences and disorders have not yet incorporated these individual differences into childhood speech/language development. This article reviews three sources of evidence in a comprehensive body of research aligning with three main themes: (a) associations between musical rhythm and speech/language processing, (b) musical rhythm in children with developmental speech/language disorders and common comorbid attentional and motor disorders, and (c) individual differences in mechanisms underlying rhythm processing in infants and their relationship with later speech/language development. In light of converging evidence on associations between musical rhythm and speech/language processing, we propose the Atypical Rhythm Risk Hypothesis, which posits that individuals with atypical rhythm are at higher risk for developmental speech/language disorders. The hypothesis is framed within the larger epidemiological literature in which recent methodological advances allow for large-scale testing of shared underlying biology across clinically distinct disorders. A series of predictions for future work testing the Atypical Rhythm Risk Hypothesis are outlined. We suggest that if a significant body of evidence is found to support this hypothesis, we can envision new risk factor models that incorporate atypical rhythm to predict the risk of developing speech/language disorders. Given the high prevalence of speech/language disorders in the population and the negative long-term social and economic consequences of gaps in identifying children at-risk, these new lines of research could potentially positively impact access to early identification and treatment.

## 1 | INTRODUCTION

Developmental speech/language disorders have a high prevalence (3–16%) in the population (National Academies of Sciences, Engineering, and Medicine, 2016) but many of these cases are identified late or are not identified at all. For example, epidemiological approaches involving screening large numbers of children consistently show prevalence rates of 7–8% for developmental language disorder (DLD; Tomblin et al., 1997), but only parents of a quarter of these children were aware their child had a speech or language problem. These low identification rates are relevant because speech/language disorders cause life-long difficulties in academic, social, and economic domains (Baker & Ireland, 2007; Beitchman, Nair, Clegg, & Patel, 1986; Cantwell & Baker, 1987; Catts, 1993; Conti-Ramsden, Durkin, Toseeb, Botting, & Pickles, 2018; Hall & Tomblin, 1978; Hubert-Dibon, Bru, Le Guen, Launay, & Roy, 2016; Law, Rush, Schoon, & Parsons, 2009; Paul & Cohen, 1984; Rice, Sell, & Hadley, 1991). Children with dyslexia and DLD are also far more likely to enter into the juvenile justice system (Snow, 2019). Importantly, long-term consequences could be attenuated with more efficient and earlier identification of the disorders and earlier intervention (Bowyer-Crane et al., 2008; Roberts & Kaiser, 2015; Snowling, 2013).

Recent work has highlighted the need to facilitate the identification of speech- and language-related developmental disorders and improve the design of early intervention by exploring risk factors in population-based samples (Raghavan et al., 2018). Converging evidence supports comorbidities among different speech/language disorders (e.g., Dyslexia and DLD; Bishop & Snowling, 2004; Catts, Adlof, Hogan, & Weismer, 2005) and also between speech/language disorders and motor disorders (e.g., developmental coordination disorder [DCD]; Kaplan, Dewey, Crawford, & Wilson, 2001; Scabar, Devescovi, Blason, Bravar, & Carrozzi, 2006; Selassie, Jennische, Kyllerman, Viggedal, & Hartelius, 2005; Zwicker, Missiuna, & Boyd, 2009) or attentional disorders (e.g., attention deficit hyperactivity disorder [ADHD], Box 1; Donaher & Richels, 2012; Kaplan et al., 2001; Kovac, Garabedian, Du Souich, & Palmour, 2001; Mueller & Tomblin, 2012; Redmond, 2016; Selassie et al., 2005; Westerlund, Bergkvist, Lagerberg, & Sundelin, 2002; Zwicker et al., 2009). This research suggests that it is unusual to have discrete, categorical developmental disorders, and that it may be more efficient to search for underlying deficits that can be identified across disorders in large samples of children. In accordance with these results, the possibility of the *transdiagnostic* approach has arisen in research, diagnosis and treatment of disorders (Mareva & Holmes, 2019).

In the current paper, we propose that atypical rhythm might be one of the underlying risk factors that has common biological underpinnings with, and may lead to, co-morbid impairments in speech/language processing. This hypothesis will be referred to as the *Atypical Rhythm Risk Hypothesis*. For the purpose of the Atypical Rhythm Risk Hypothesis, we define *atypical rhythm* as a general construct capturing any/all of the following terms: Impairments in rhythm/beat/ meter sensitivity, significantly weaker than normal rhythm ability/skill, poor dynamic attending, beat deafness (Sowiński & Dalla Bella, 2013), or time-based amusia (Peretz & Vuvan, 2017). Atypical rhythm can be classified by poor performance on any implicit or explicit perception or production task of rhythm or timing, such as rhythm discrimination, interval discrimination, rhythm, beat, or meter processing, and synchronization or entrainment. Atypical rhythm may also be described as a rhythm impairment or a rhythm disorder. While the underlying neural mechanisms giving rise to different manifestations of typical and atypical rhythm are of great interest (Fiveash, Bedoin, & Tillmann, submitted), in this article, we will primarily focus on the clinical significance of common biological risk factors across different manifestations of atypical rhythm.

In light of recent genetic epidemiological approaches showing shared genetic architecture between related, but clinically distinct traits (e.g., common heritability across many different brain disorders; Anttila et al., 2018), new avenues for the exploration of common risk factors, such as atypical rhythm, can now be pursued and eventually expanded into the genetic domain. There are several genetic and environmental risk factors proposed for speech/language disorders, and presumably, there are other risk factors still to be discovered (e.g., for a multifactorial view of language disorders focusing on dyslexia: Bishop, 2015; focusing on DLD: Bishop et al., 2017). In line with this multifactorial view of language disorders, we propose that, within a pool of risk factors, a generalized rhythm/timing deficit may interact with other genetic or environmental risk factors. We here synthesize evidence linking rhythm to speech/language development and propose an overarching theoretical framework as groundwork for testing the Atypical Rhythm Risk Hypothesis.

If future research supports the Atypical Rhythm Risk Hypothesis, new possibilities for incorporating rhythm tests into clinical practice may open up. For example, using atypical rhythm as a risk factor for the development of speech and language disorders could be beneficial for improving early identification of these disorders. Atypical timing skills can be measured with tasks targeting musical rhythm perception, which can be assessed earlier in development (around 7–10 months, see Kalashnikova, Goswami, & Burnham, 2019, or already in 2- to 3-day-old newborns, see Winkler, Haden, Ladinig, Sziller, & Honing, 2009) than primary symptoms of speech/language disorders (e.g., atypical reading in dyslexia and atypical expressive grammar in DLD that can be assessed at preschool age at the earliest, or atypical speech production in stuttering that can be assessed from age two at the earliest). In addition, rhythm tasks could be included in the language screening of preschool-age children. To develop the hypothesis of musical rhythm processing at infancy and early childhood as a risk factor for speech/language disorders, we will synthesize different lines of research investigating whether (a) musical rhythm and speech/language skills are associated, (b) rhythm is impaired in speech/language disorders and common comorbid attentional and motor disorders, and (c) individual differences in mechanisms underlying rhythm processing skills at infancy are related to language development and the presence/absence of speech/language disorders in childhood. We will also frame this hypothesis within the larger epidemiological literature that has recently experienced a series of methodological advances allowing for large-scale testing of shared underlying biology across clinically distinct disorders. We then outline a series of predictions for future work testing the Atypical Rhythm Risk Hypothesis.

## 2 | PERCEPTUAL AND NEURAL MECHANISMS OF HUMAN RHYTHM PROCESSING ABILITY AND THEIR RELATION TO HIGHER-LEVEL LANGUAGE PROCESSES

Temporal regularities are present at multiple hierarchical levels in both music and speech. In the domain of music, this regularity is more salient as temporal intervals of the underlying beat are isochronous, whereas in speech there is a higher variability in intervals (e.g., also referred to as quasi-periodic: Peelle & Davis, 2012). When listening to music, a *basic* frequency serves as a temporal organizer; it is referred to as the pulse or beat and typically falls between 1 and 2 Hz (see London, 2004). Strong and weak beats are grouped into a hierarchical metrical structure, which is a cognitive construct of the listener (Lerdahl & Jackendoff, 1983). In spoken language, the rhythm of speech is carried by the so-called amplitude envelope, which captures information about duration, rhythm, tempo, and stress of speech (Goswami, 2019; Kotz, Ravignani, & Fitch, 2018; Myers, Lense, & Gordon, 2019). When the amplitude envelope is degraded, speech can become unintelligible (Ghitza, 2012). Similarly to musical rhythm, groupings of strong and weak accented speech events (such as stressed and unstressed syllables) form metrical structures. These stress patterns play a role both in language acquisition (Bernard & Gervain, 2012; de Carvalho, Dautriche, & Christophe, 2016; Dupoux, Pallier, Sebastian, & Mehler, 1997; Gervain & Werker, 2013; Jusczyk, Cutler, & Redanz, 1993) and speech processing (Bion, Benavides-Varela, & Nespor, 2011; Dilley & McAuley, 2008). At this point, we would like to emphasize that when we use the term rhythm processing in the paper both in relation to music and speech, we are also referring to the processing of beat and metrical structures of the stimuli as an aspect of rhythm processing.

Based on accumulating evidence in recent research (summarized below), several theories have outlined shared underlying processes for musical rhythm and speech processing: The dynamic attending theory (DAT; Jones, 1976, 2016; Jones & Boltz, 1989; Large & Jones, 1999), which inspired many of the later theories; the temporal sampling framework (TSF; Goswami, 2011, 2018); the sound envelope processing and synchronization and entrainment to pulse hypothesis (SEP; Fujii & Wan, 2014); the precise auditory timing hypothesis (PATH; Tierney & Kraus, 2014); and the OPERA hypothesis (Patel, 2011, 2012). Fiveash et al. (submitted) highlight three common elements in these theories and propose their combination as crucial for music rhythm and speech processing. (a) All theories emphasize the role of *fine-grained auditory processing* (precise, low-level processing of the acoustic signal) in music and speech as a necessary element underlying perception and transfer effects between domains. (b) *Neural oscillations* and their entrainment to the auditory stimuli play a role in structural processing (including hierarchical processing), temporal integration, and prediction of music and speech signals. According to the DAT (Jones, 2019; Large & Jones, 1999), endogenous brain oscillations synchronize with external regularities and predictable cues (e.g., beat or stress), help to structure the auditory input, and focus attention to important elements of the auditory stimulus and its presentation over time (see also Ghitza, 2011; Giraud & Poeppel, 2012; Peelle & Davis, 2012). (c) The role of *sensorimotor coupling* both in music rhythm and speech/language processing is included in several of these theories. The involvement of motor functions is not surprising in the case of speaking or when moving to music, but interestingly, motor areas are consistently found to be activated during the perception of music (Chen, Penhune, & Zatorre, 2008; Fujioka, Trainor, Large, & Ross, 2012; Gordon, Cobb, & Balasubramaniam, 2018; Grahn & Brett, 2007; Stephan, Lega, & Penhune, 2018) and speech (Glanz et al., 2018; Möttönen, Dutton, & Watkins, 2013; Wilson, Saygin, Sereno, & Iacoboni, 2004), even in the absence of overt movement.

In line with the central role of these three processes, several studies have shown overlapping brain activations in auditory and motor cortices for both musical rhythm and speech/language processing (Chen, Zatorre, & Penhune, 2006; Grahn & Brett, 2007; Keitel, Benwell, Thut, & Gross, 2018; Kotz et al., 2018). Kotz, Schwartze, and Schmidt-Kassow (2009) outlined a network involving frontal (dorsolateral prefrontal cortex), supplementary motor area (SMA) and basal ganglia regions (the pre-SMA-basal ganglia circuit). They suggest that this circuitry is involved in the processing of predictable sensory cues, such as beat in music or word stress/linguistic meter in speech. The authors also emphasize the role of neural oscillations and propose that the pre-SMA-basal ganglia circuit regulates the synchronization of neural oscillations with auditory stimuli and therefore plays a crucial role in predicting when the next event will occur in a sequence. Although cortical oscillations can be reliably measured in humans, oscillatory activity originating from subcortical structures, such as the basal ganglia, cannot be easily isolated. However, primate research has provided evidence that oscillations in the beta frequency band (which have been shown to play a crucial role in rhythm processing: Zanto, Large, Fuchs, & Kelso, 2005) originate from the basal ganglia, suggesting a similar function in humans (Merchant & Bartolo, 2018).

Another line of research emphasizes the shared nature of structural and hierarchical processing in language and music—including rhythm processing. According to Lashley (1951), language and music are both made up of sub-elements that need to be correctly ordered in the temporal domain. The organization of the sub-elements can be described by hierarchical, tree-like structures, in which lower levels are incorporated by higher levels, and are ordered according to specific rules (Fitch, 2017). Fitch and Martins (2014) proposed that the emergence of tree-like syntax in the grammatical structure of language in early humans was an evolutionary turning point that might also have coincided with the emergence of the metrical structure of rhythm in human musicality. In line with this proposal, hierarchical structures in language and music seem to be processed using similar cognitive and neural mechanisms (Patel, 2003, 2008). Although most of the studies investigating shared structural processing between music and language have focused on harmonic syntax or music processing in general (Fiveash, McArthur, & Thompson, 2018; Fiveash & Pammer, 2014; Herdener et al., 2014; Hoch, Poulin-Charronnat, & Tillmann, 2011; Jentschke & Koelsch, 2009; Jentschke, Koelsch, Sallat, & Friederici, 2008; Koelsch, Gunter, Wittfoth, & Sammler, 2005; Kunert, Willems, & Hagoort, 2016; Slevc, Rosenberg, & Patel, 2009; Steinbeis & Koelsch, 2008), recent evidence suggests some associations between linguistic and rhythmic syntax as well (Sun, Liu, Zhou, & Jiang, 2018).

Hierarchically organized neural oscillations, emphasized by several theories of musical rhythm and speech processing, might play a crucial role in the processing of hierarchically organized syntactic structures as well. According to the Metric Binding Theory by Jones (2019), it is the internal entrainment of multiple nested neural oscillators and their *binding* that support meter processing and enhances temporal predictions. It is possible that the same process might extend to language and allow for higher-level structure learning and processing. This hypothesis is supported by studies showing that neural oscillations entrain not only to physically marked beats and stressed syllables, but also to higher-level structures both in music (e.g., the metrical structure; Nozaradan, Peretz, Missal, & Mouraux, 2011) and language (e.g., syntactic structure; Ding, Melloni, Zhang, Tian, & Poeppel, 2015), which are not necessarily physically present in the signal (Fiveash et al., 2020; Tal et al., 2017). Efficient entrainment, defined here as the precise phase-locking of neural oscillations at the appropriate frequency, to higher-level structures in musical rhythm and language may also lead to improved prediction skills, possibly through attention allocation (e.g., Large & Jones, 1999; Schmidt-Kassow & Kotz, 2009). Entrainment and increased attention to important parts of the signal may not only facilitate *temporal* predictions (e.g., predicting *when* something will happen; *predictive timing*, Friston, 2005), but also lead to better predictions of *what* will happen next (*predictive coding*, Friston, 2005; Jones & Boltz, 1989; Koelsch, Vuust, & Friston, 2019). The predictions developed for what and when of incoming input allow for faster and more efficient processing of these events and their underlying structures, whether musical or linguistic. The possibility of such a link between predictive skills in rhythm and language is supported by preliminary evidence showing that children who are impaired in tapping tasks are also worse at making structure-based morpho-syntactic predictions in language (Persici, Stucchi, & Arosio, 2019).

**FIGURE 1:**
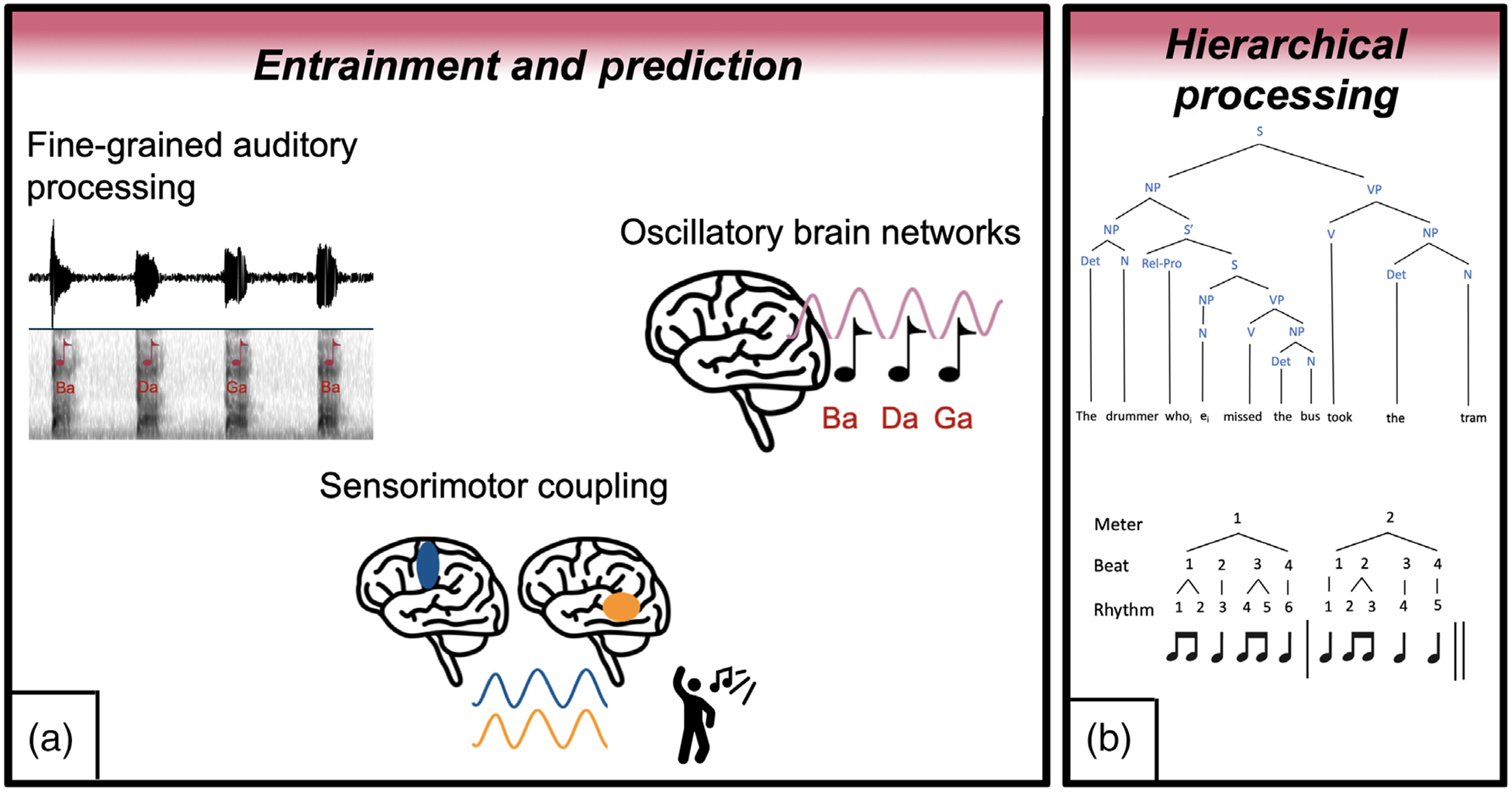
Shared underlying mechanisms for musical rhythm and speech/language processing. (a) Models of underlying mechanisms for musical rhythm and speech/language processing emphasize the role of fine-grained auditory processing, oscillatory brain networks and sensorimotor coupling (Fiveash et al., submitted). (b) Another line of literature emphasizes the shared role of processing of hierarchical structures in both musical rhythm and syntactic processing (e.g., Fitch & Martins, 2014; bottom figure is adapted from Heard & Lee, 2020)

The research findings reviewed above suggest that a shared network underlies musical rhythm and speech/language processing that supports the processing of surface-level features of musical rhythm and speech as well as the processing of syntactic structures in musical rhythm and language (Figure 1). In the following sections, we summarize evidence for associations between musical rhythm and speech/language processing in typical and atypical populations.

## 3 | INDIVIDUAL DIFFERENCES: A SYNTHESIS OF RESEARCH INVESTIGATING ASSOCIATIONS BETWEEN RHYTHM AND SPEECH/LANGUAGE IN TYPICALLY DEVELOPING INDIVIDUALS

**FIGURE 2:**
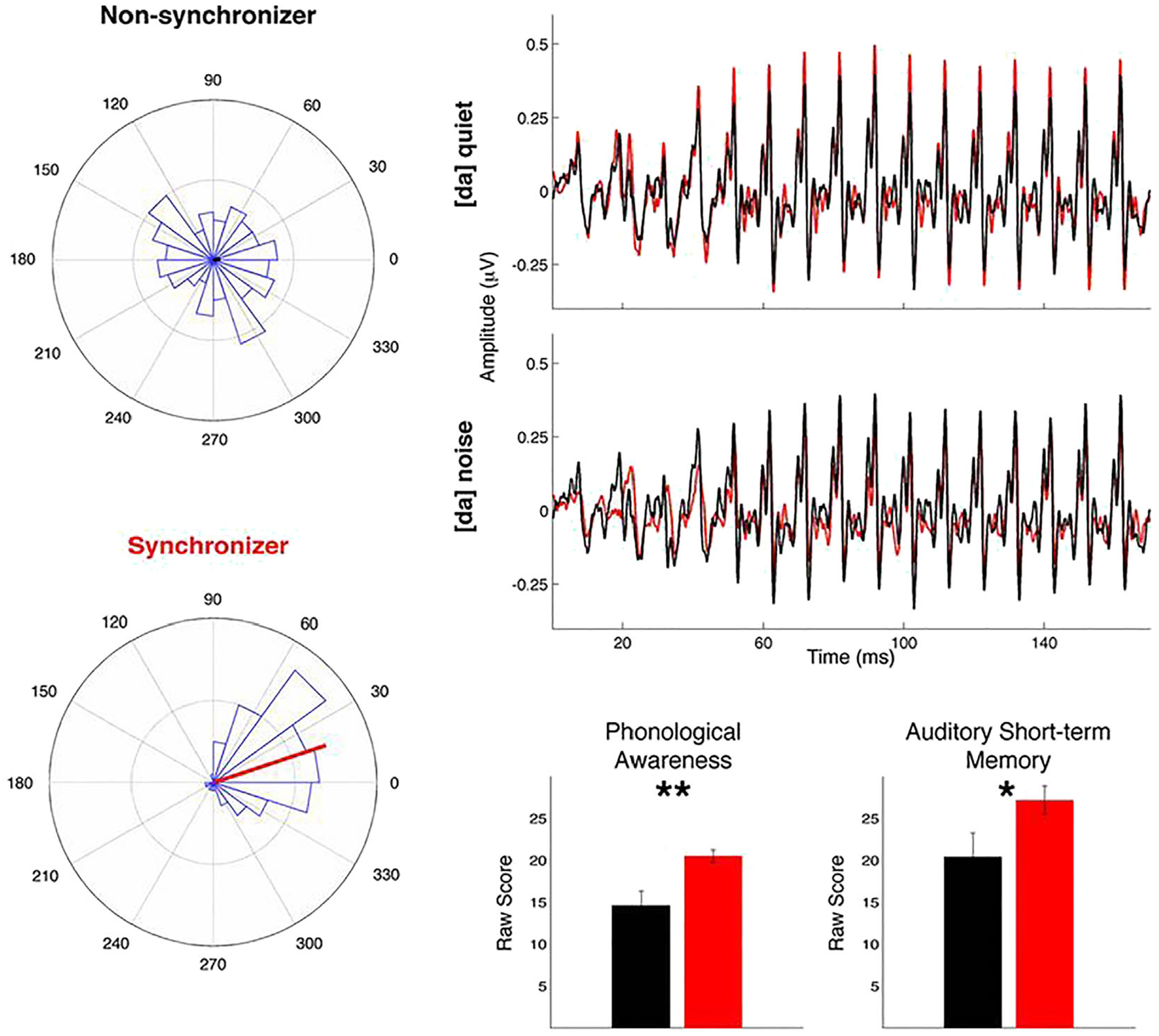
Rhythm production ability and early literacy skills. Convergent evidence across data acquired with various methods, in support of associations between rhythm skills and language in preschool-aged children (Reprinted with permission from Woodruff Carr, White-Schwoch, Tierney, Strait, and Kraus (2014)). Children that performed well on a musical beat synchronization task (here called *synchronizers*, in red, shown on the rose plots on left to have better drumming accuracy) encoded speech more efficiently (top right) and show significantly better phonological awareness than their *non-synchronizer* peers (bottom right). Synchronizers also performed better on a sentence repetition task (it is important to note that sentence repetition/imitation tasks not only require auditory perception and short-term memory but also reflect deeper access to the grammatical structure of language: see Klem et al., 2015)

Overlapping neural processes underlying musical rhythm and speech/language abilities are supported by a large body of literature showing associations between individual differences in language and rhythm skills (Anvari, Trainor, Woodside, & Levy, 2002; Degé, Kubicek, & Schwarzer, 2015; Douglas & Willatts, 1994; Gordon et al., 2015; Grube, Kumar, Cooper, Turton, & Griffiths, 2012; Holliman, Wood, & Sheehy, 2010; Magne, Jordan, & Gordon, 2016; Moritz, Yampolsky, Papadelis, Thomson, & Wolf, 2013; Ozernov-Palchik, Wolf, & Patel, 2018; Strait, Hornickel, & Kraus, 2011). For instance, beat synchronization and early literacy as well as spoken language skills are strongly linked (see Figure 2 showing data from Woodruff Carr et al., 2014). In addition, there is ample evidence of better performance on various language tasks after rhythm/music training in the typically developing population (Degé & Schwarzer, 2011; Linnavalli, Putkinen, Lipsanen, Huotilainen, & Tervaniemi, 2018; Patscheke, Degé, & Schwarzer, 2016; Rautenberg, 2015; Taub & Lazarus, 2012; Zhao & Kuhl, 2016). Moreover, several studies have found a short-term facilitating effect of regular rhythm on subsequent grammar task performance in typically developing children (Ladányi, Lukács, & Gervain, submitted; Bedoin, Brisseau, Molinier, Roch, & Tillmann, 2016; Canette et al., 2020; Chern, Tillmann, Vaughan, & Gordon, 2018; Przybylski et al., 2013). In addition, better speech/language skills, such as more efficient speech processing and word segmentation, have been reported for musicians compared to non-musicians (Brod & Opitz, 2012; François, Jaillet, Takerkart, & Schön, 2014; Marie, Magne, & Besson, 2011; Musacchia, Sams, Skoe, & Kraus, 2007; Sares, Foster, Allen, & Hyde, 2018; Zuk et al., 2013), although this advantage could originate from other differences between musicians and non-musicians beyond differences in rhythm skills. It is also important to note that individual differences in musical *ability* or aptitude in adults predict speech perception task performance beyond musical training (Mankel & Bidelman, 2018). Interestingly, evidence extends beyond surface-level auditory characteristics of speech and to deeper, hierarchically structured syntactic processing of language (Gordon, Jacobs, Schuele, & McAuley, 2015; Politimou, Dalla Bella, Farrugia, & Franco, 2019; Woodruff Carr et al., 2014). A complete review of correlations between rhythm and speech/language skills in typical development is beyond the scope of the present paper (see Fiveash et al., submitted).

## 4 | ATYPICAL RHYTHM IN CHILDREN WITH ATYPICAL SPEECH/LANGUAGE DEVELOPMENT

Associations between rhythm and speech/language processing are strongly supported by recent research demonstrating that children with speech/language developmental disorders (e.g., dyslexia, DLD, stuttering), as well as children with speech/language impairments as co-morbid deficits in other developmental disorders (e.g., DCD, ADHD), often exhibit underlying timing deficits that could be contributing to the symptomatology within each pathology. In each of these disorders, research has revealed some evidence for associated timing impairments, even though specifics of these impairments differ (see a summary of this research in Table 1). Considering that there are high levels of comorbidity between disorders (Bishop & Snowling, 2004; Catts et al., 2005; Donaher & Richels, 2012; Kaplan et al., 2001; Kovac et al., 2001; Mueller & Tomblin, 2012; Redmond, 2016; Scabar et al., 2006; Selassie et al., 2005; Westerlund et al., 2002; Zwicker et al., 2009), it is likely that there are shared impairments in underlying neural mechanisms across different pathologies. We will return to possible etiologies of co-morbidities across these disorders in Section 6; here we focus on atypical rhythm in several highly prevalent developmental speech/language disorders. Together with Fiveash et al. (submitted), we suggest that common deficits in timing may be largely related to impaired fine-grained auditory processing, impaired tracking of rhythms via neural oscillations, and impaired sensorimotor coupling in the brain. We further propose that impaired hierarchical processing could result in both impaired processing of rhythmic structures and syntactic processing of language. Impairment in one or more of these underlying mechanisms appears to be associated with atypical speech/language processing, rhythm processing and/or motor impairments. In addition, we need to consider environmental factors and genetic family history, as further discussed below.

### 4.1 | Atypical rhythm in dyslexia

Recent research has shown that children with dyslexia are impaired in comparison to typically developing children in rhythm perception and production tasks. According to the Temporal Sampling Framework (TSF; Goswami, 2011), many of the processing deficits observed in dyslexia may be accounted for by inefficient entrainment of brain oscillations to sensory input, which in turn is theorized to affect not only rhythm processing but also phonological processing as well as other aspects of language processing. Studies investigating neural entrainment in individuals with dyslexia support this hypothesis by showing deficits in synchronization to the speech envelope (Leong & Goswami, 2014; Molinaro et al., 2016; Power et al., 2016) regardless of the language spoken (e.g., English: Goswami et al., 2010; English and Hungarian: Surányi et al., 2009; Chinese: Wang et al., 2012) and atypical neural entrainment to nonspeech stimuli compared to controls (Cutini et al., 2016; Frey et al., 2019). Further studies have shown impaired beat synchronization in individuals with dyslexia (Colling et al., 2017; Overy et al., 2003; Thomson & Goswami, 2008). Relatedly, rhythm, language, and reading skills are correlated: Individuals with dyslexia who show weaker performance in rhythm perception and production tasks also show weaker phonological awareness (Flaugnacco et al., 2014; Forgeard, Winner, Norton, & Schlaug, 2008; Goswami et al., 2010; Huss et al., 2011; Lee et al., 2015; Thomson & Goswami, 2008) and reading skills (Dellatolas, Watier, Le Normand, Lubart, & Chevrie-Muller, 2009; Flaugnacco et al., 2014; Goswami et al., 2002, 2010; Goswami, Huss, et al., 2013; Goswami, Mead, et al., 2013; Huss et al., 2011; Muneaux et al., 2004; Thomson & Goswami, 2008). Individuals with dyslexia also show impaired processing of rise-time information, and this deficit has been linked to inefficient entrainment of neural oscillations to the speech stream (Goswami et al., 2016; Huss et al., 2011; Leong, Hämäläinen, Soltész, & Goswami, 2011_S1_Reference170; Thomson et al., 2006). Deficits in neural entrainment to higher-level structures throughout development may also result in impaired hierarchical processing skills. Interestingly, recent research suggests that children with dyslexia perform below age-matched peers in tasks that require the use of morphological information to predict incoming material (Persici et al., 2019).

These phonological and rhythmic deficits do not appear to fully recover later in development, though some studies suggest that deficits in the adult population may be constrained to the type of measure used (Leong & Goswami, 2014). Adults with dyslexia show significantly weaker synchronization and beat perception skills as compared to adults with typical development (Pasquini et al., 2007; Thomson et al., 2006), and exhibit impaired low-frequency neural entrainment, regardless of whether speech (Molinaro et al., 2016) or nonspeech (Hämäläinen et al., 2012; Lizarazu et al., 2015) stimuli were used. As in children, this temporal processing deficit in keeping time with an external stimulus is particularly disrupted at 2 Hz (Soltész et al., 2013), a frequency that is also important for speech perception, as it corresponds to the accented syllabic rate. Musical training may reduce these processing deficits in individuals with dyslexia, as it has been shown that musicians with dyslexia have better auditory temporal processing than nondyslexics (Bishop-Liebler, Welch, Huss, Thomson, & Goswami, 2014) and better amplitude information processing skills than nonmusicians with dyslexia (Zuk et al., 2017).

Building on these observed connections, a few studies have aimed to apply rhythm training approaches in children with dyslexia and found improved language- and reading-related skills after training (Bonacina, Cancer, Lanzi, Lorusso, & Antonietti, 2015; Flaugnacco et al., 2015; Habib et al., 2016; Overy, 2000; Thomson, Leong, & Goswami, 2013). Interestingly, even a short presentation of rhythmic musical primes improves grammatical processing of subsequently presented sentences in children (Przybylski et al., 2013) and adults (Canette et al., 2019) with dyslexia. These results further support the hypothesis that rhythm and language processing are related, and show that music rhythm training in the long-term and rhythm stimulation in the short-term may be useful approaches to improve language skills in addition to more traditional language-centered therapeutic methods (Schön & Tillmann, 2015).

### 4.2 | Atypical rhythm in developmental language disorder

Children with DLD show difficulties in both speech and music rhythm processing (Bedoin et al., 2016; Cumming et al., 2015; Sallat & Jentschke, 2015; Weinert, 1992). They have weaker synchronization skills than controls when asked to tap with the beat (Corriveau & Goswami, 2009; Cumming et al., 2015), though synchronization deficits are not observed in all studies (Zelaznik & Goffman, 2010), or for all types of tapping tasks (Vuolo et al., 2017, in which differences in synchronization skills between typically developing and children with DLD were only found when participants were asked to use both hands in a clapping task compared to just one hand). Recent studies have demonstrated the presence of deficits in amplitude envelope and rise-time information processing for children with DLD (Corriveau et al., 2007; Goswami et al., 2016; Richards & Goswami, 2015). Impaired sensitivity to amplitude rise-time has been associated with poor performance on language and literacy measures (such as vocabulary attainment, phonological awareness, and reading; Corriveau et al., 2007) and speech stress processing (Cumming et al., 2015; Richards & Goswami, 2015). Similar patterns in children with DLD and dyslexia led Goswami to extend the TSF to DLD, suggesting shared underlying impairments across disorders (Goswami et al., 2016).

Several studies also reported difficulties in prosody processing in children with DLD compared to TD children (Fisher, Plante, Vance, Gerken, & Glattke, 2007; Richards & Goswami, 2019; Sabisch et al., 2009; Wells & Peppé, 2003), whereas others report intact prosody perception in DLD (Goffman, 2004). Weinert (1992) found that the ability to take advantage of prosodic information in children with DLD was associated with their performance on a rhythm discrimination task, suggesting that impaired processing of prosody and rhythm may be caused by an underlying impairment in the processing of temporal cues.

Similarly to dyslexia, the presentation of a regular rhythmic prime enhances subsequent grammatical sentence judgments in children with DLD compared to both irregular primes (Ladányi et al., submitted; Przybylski et al., 2013) and neutral non-musical auditory primes (Bedoin et al., 2016), supporting the hypothesis that rhythm and language processing are related and suggested that using rhythm in the therapy of children with DLD might facilitate speech/language therapy.

### 4.3 | Atypical rhythm in stuttering

**FIGURE 3:**
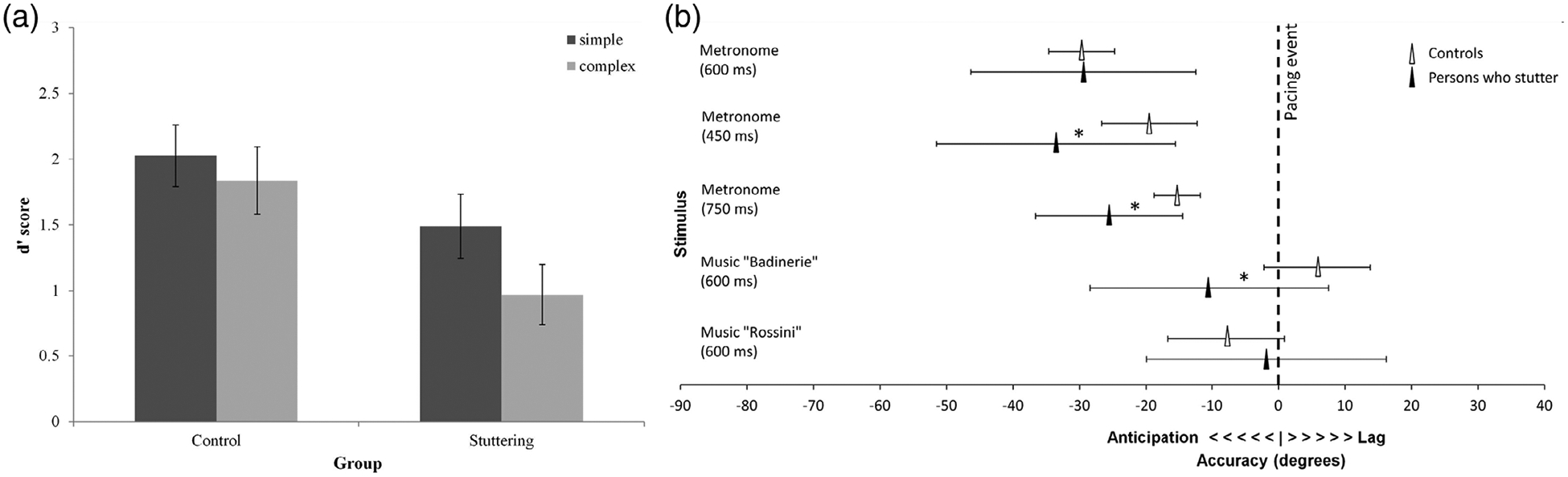
Rhythm perception and production difficulties in children with stuttering. (a) Children who stutter show impaired rhythm perception performance compared to TD children on a task requiring discrimination of simple and complex musical rhythmic sequences (Wieland et al., 2015). (b) Children and adolescents who stutter are less accurate than TD peers on synchronization tests, both tapping to a metronome at certain rates and tapping to the beat in music (Falk et al., 2015). Both figures are reprinted with permission from the original papers

Recent research has suggested that speech dysfluency in stuttering is associated with impaired sensorimotor coupling (Chang et al., 2016; Hickok, Houde, & Rong, 2011) and a disruption to the production of timing cues from the basal ganglia (Alm, 2004; Toyomura et al., 2011). Individuals who stutter tend to be impaired in several types of rhythmic tasks, including unpaced tapping, which relies on internal time keeping (Olander et al., 2010). In addition, they show weaker synchronization to an external stimulus (Falk et al., 2015), and poorer rhythm discrimination (Wieland et al., 2015; see Figure 3) than typically developing peers. Impaired predictive timing via sensorimotor coupling has been suggested as the underlying cause of the rhythm deficits reported for individuals who stutter (Hickok et al., 2011). Interestingly, it has been shown that the addition of external auditory stimulation can attenuate stuttering, potentially because it provides an external rhythmic cue to compensate for the impaired internal time keeping (Toyomura et al., 2011). Singing also enhances fluency in speech, likely by regulating the temporal structure of the words (Falk, Maslow, Thum, & Hoole, 2016; Glover, Kalinowski, Rastatter, & Stuart, 1996; Wan, Rüber, Hohmann, & Schlaug, 2010).

### 4.4 | Atypical rhythm in other speech disorders

Although several speech disorders are differentiated in the literature beyond stuttering (speech-sound disorders including articulation/phonological disorder, dysarthria, and childhood apraxia of speech, and voice disorders), most have a known physiological cause (e.g., cleft palate, impaired laryngeal structures, or brain trauma). At the same time, the underlying cause of some forms of articulation/phonological disorder and childhood apraxia is unknown, and they may have partly shared etiology and comorbidities with the other speech/language disorders discussed here. Articulation and phonological sequencing, which requires timing and motor skills, are often impaired in these children. Given the timing demands of sequencing, we believe it would be of great interest to investigate atypical rhythm in these populations. We are aware of one study that explored rhythm processing in individuals with speech difficulties (Alcock, Passingham, Watkins, & Vargha-Khadem, 2000). The authors investigated nine individuals (children and adults) belonging to the same family (KE family) showing both expressive and receptive speech and language impairments together with difficulties with nonverbal oral movements, linked to rare variants in the gene *FOXP2*. Affected family members performed worse both on rhythm perception and production tasks compared to control participants. Future work is needed to explore rhythm across different motor speech disorders.

### 4.5 | Atypical rhythm in developmental coordination disorder

In contrast to numerous studies investigating the motor circuitry involved in musical rhythm processing in typically developing individuals (Merchant, Grahn, Trainor, Rohrmeier, & Fitch, 2015), and atypical rhythm processing in individuals with Parkinson’s Disease (Grahn & Brett, 2009; Harrington, Haaland, & Hermanowitz, 1998; O’Boyle, Freeman, & Cody, 1996),^1^ only a few studies have examined rhythm processing in children with DCD, a disorder characterized by impaired motor abilities, especially related to postural control, motor learning, and sensorimotor coordination that affect quality of life (Zwicker et al., 2009). Children with DCD show poorer synchronization to an external beat compared to typically developing children in synchronization tasks (Rosenblum & Regev, 2013), and children with both ADHD and DCD show even poorer synchronization compared to children with just ADHD or matched controls (Puyjarinet et al., 2017). However, all of these studies investigated performance in rhythm production tasks, which may be easily affected by inherent motor coordination deficits. Only Trainor et al. (2018) have investigated auditory timing with perceptual tasks in DCD; their first behavioral and neuroimaging evidence suggest that auditory perceptual timing (measured with duration and rhythmic discrimination tasks) may also be impaired in this population. Interestingly, motor impairments in children with DCD have also been associated with difficulties in language processing (Mirabella et al., 2017). Future research should now investigate more specifically the potential timing and/or perception deficits in DCD as well as whether and how the impairments in timing might be related to language processing skills in cases of DCD with atypical language development.

### 4.6 | Atypical rhythm in attention deficit hyperactivity disorder

Recent work suggests that both children and adults with ADHD show poorer performance in paced and unpaced tapping and body movement synchronization tasks compared to controls (Amrani & Golumbic, 2019; Carrer, 2015; Hove et al., 2017; Noreika, Falter, & Rubia, 2013; Slater & Tate, 2018; Valera et al., 2010; Zelaznik et al., 2012), especially when synchronization requires beat extraction (Puyjarinet et al., 2017). Though it is difficult to disentangle the role of more generalized attentional deficits from deficits in temporal processing (and in particular, temporal attention and dynamic attending), this emerging literature points to difficulties with both synchronization and internal time-keeping in ADHD (see Falter & Noreika, 2014, for a review). Future work in larger ADHD samples with a variety of rhythm tasks is needed to tease apart various dimensions of rhythm processing, their potential deficits in ADHD, and how they relate to domain-general attentional deficits.

## 5 | CAN ATYPICAL RHYTHM AT INFANCY PREDICT ATYPICAL SPEECH/LANGUAGE DEVELOPMENT?

The reported associations between rhythm and speech/language processing as well as atypical rhythm processing in speech/language disorders lead to the hypothesis that atypical musical rhythm processing skills at infancy could be used as a risk factor for speech/language disorders. This type of approach has been employed by Kalashnikova et al. (2019), who showed longitudinal evidence for a predictive relationship between temporal processing (measured with amplitude rise time) at infancy and oral language development. Infants’ performance on an amplitude envelope rise time discrimination task at 7–10 months of age-predicted children’s performance on vocabulary tests at three years of age. To examine this potential predictive relationship between temporal processing and language development further, we first summarize research about rhythm processing in infants to explore whether infants reliably process rhythm and whether it can be measured experimentally. Then, we discuss work exploring individual differences in underlying rhythm processing mechanisms and their relationship with later language development.

Experimental evidence suggests that rhythm processing starts to develop very early in life. A few infant studies showing behavioral (Hannon & Trehub, 2005; Phillips-Silver & Trainor, 2007; Zentner & Eerola, 2010) and electrophysiological (Cirelli, Spinelli, Nozaradan, & Trainor, 2016) evidence indicate that infants and newborns (Winkler et al., 2009) process rhythmic regularities (i.e., beat, meter) in musical stimuli. Infants are also sensitive to the rhythmic cues of speech. Newborns can discriminate between languages from different rhythmic categories (Mehler et al., 1988; Nazzi, Bertoncini, & Mehler, 1998; Ramus, 2000) and discriminate words with different patterns of lexical stress (Sansavini, Bertoncini, & Giovanelli, 1997). Further, infants exploit lexical stress for word segmentation (Dupoux et al., 1997; Jusczyk, 1999) and phrasal level prosody for grammar acquisition (Bernard & Gervain, 2012; de Carvalho, Dautriche, Lin, & Christophe, 2017; de Carvalho et al., 2016; Gervain, 2018; Gervain & Werker, 2013; see electrophysiological data for infant’s sensitivity to speech rhythm in Kalashnikova, Peter, Di Liberto, Lalor, & Burnham, 2018), suggesting an important role of speech rhythm in language development. Based on the research reviewed thus far, we suggest that infants may use the same mechanisms for processing rhythm in the two domains. Future research is needed to compare the benefits of measuring the processing of music rhythm versus speech rhythm in infants in order to predict speech/language development, as each domain has a unique set of constraints and advantages. However, the temporal regularity of music rhythm makes it a useful tool to measure neural entrainment in infants, especially under noisy testing conditions.

Taken together, these studies suggest that rhythm processing is functional from birth, and rhythm skills can be measured both behaviorally and physiologically. Aiming to use atypical rhythm as a risk factor for speech/language disorders also requires knowledge of whether infants show individual differences in rhythm processing and importantly, whether these differences might be related to later speech/language development as well as the presence/absence of speech/language disorders. Although we did not find any studies exploring these questions by measuring musical rhythm processing, numerous studies have investigated infants’ abilities related to fine-grained auditory processing—one of the shared fundamental aspects of rhythm sensitivity that we outlined in the *Introduction* based on Fiveash et al. (submitted). Neural entrainment of oscillations and sensorimotor coupling has been investigated by some studies, but to the best of our knowledge, no studies have investigated the relationship between rhythm and hierarchical processing of syntactic structures in infants. We are only aware of studies exploring rhythm-related mechanisms at infancy in relation to dyslexia or DLD; therefore, we discuss these results below. While we do not cover stuttering, DCD or ADHD in the remainder of this section, similar logic could be applied to testing the developmental precursors of rhythm processing and their predictive strength for language development in these populations.

### 5.1 | Fine-grained auditory processing

The majority of studies exploring fine-grained auditory processing and its relationship to later speech/language development have investigated infants with a family history of language disorders (i.e., dyslexia and/or DLD). Several studies have shown altered neural responses to auditory stimuli in infants with a family history of dyslexia both for verbal stimuli (Leppanen, Pihko, Eklund, & Lyytinen, 1999; Lohvansuu, Hämäläinen, Ervast, Lyytinen, & Leppänen, 2018; Richardson, Leppänen, Leiwo, & Lyytinen, 2003; Thiede et al., 2019; van Herten et al., 2008; van Leeuwen et al., 2006) and nonverbal stimuli (Leppänen et al., 2010; Plakas, van Zuijen, van Leeuwen, Thomson, & van der Leij, 2013; van Zuijen et al., 2012), in comparison to infants without family history of dyslexia. Infants with a family history of language or reading difficulties showed less efficient rapid auditory processing according to both behavioral and electrophysiological measures compared to children without a family history of such difficulties (Benasich & Tallal, 2002; Benasich, Thomas, Choudhury, & Leppänen, 2002; Cantiani et al., 2016; Cantiani et al., 2019; Choudhury & Benasich, 2011; Choudhury, Leppanen, Leevers, & Benasich, 2007; Raschle, Stering, Meissner, & Gaab, 2014). Multiple measures of fine-grained auditory processing at infancy were also associated with individual differences in later language and literacy development (Benasich et al., 2002; Cantiani et al., 2016, 2019; Choudhury & Benasich, 2011; Guttorm et al., 2005; Guttorm, Leppänen, Hämäläinen, Eklund, & Lyytinen, 2010; Kalashnikova et al., 2019; Leppänen et al., 2010; Lohvansuu et al., 2018; van Zuijen, Plakas, Maassen, Maurits, & van der Leij, 2013).

In light of the studies reported above, consistent differences in fine-grained auditory processing between infants with and without a family history of language disorders suggest a shared underlying biology for fine-grained auditory processing and a family history of language disorders. Phenotypic associations occur as a result of a combination of shared genetics and shared environment. Auditory processing shows a moderate to high heritability (32–74%), depending on the exact mechanism measured (Brewer et al., 2016), suggesting a strong genetic component in the phenotypic association between family history of speech/language disorders and fine-grained auditory processing. These results suggest that fine-grained auditory processing is one of the risk factors that may increase risk of language disorder depending on the interplay between this and other risk factors, such as maternal education level or perinatal circumstances (Leppänen et al., 2010; Leppänen et al., 2011).

### 5.2 | Oscillatory brain networks

We are aware of only one study investigating oscillatory brain activity in infants and its relationship to later speech/language development (Cantiani et al., 2019). In this study, oscillatory activity was measured in 6-month-old infants with or without a family history of language or reading impairment in a rapid auditory processing paradigm. The authors found a reduction in gamma power in infants with versus without a family history of language or reading difficulties, and concluded that atypical oscillatory activity might explain inefficient rapid auditory processing in infants (Heim, Friedman, Keil, & Benasich, 2011, for gamma oscillations with reduced power and attenuated phase-locking in children with impaired language or reading impairment). In addition, oscillatory measures were associated with expressive vocabulary at 20 months. These results suggest that (a) there is a phenotypic association between inefficient speech/language-related oscillatory activity and familial risk of language and reading disorders, and (b) the efficiency of oscillatory activity during auditory processing is associated with language development, although further research is needed to explore these associations. The relationship of oscillatory activity at infancy with language disorders later in school-aged children has not been investigated up to now.

### 5.3 | Sensorimotor coupling

The third shared element underlying rhythm and speech/language processing proposed by Fiveash et al. (submitted) is sensorimotor coupling. We are not aware of any studies measuring the relationship between sensorimotor coupling and language development in infants, but a few studies have explored associations between the role of motor functions in general in infants and their relationship with speech/language disorders. Atypical motor development could also serve as a risk factor for speech/language disorders, as studies show impaired fine motor skills (e.g., in a peg moving task where small pegs are placed as fast as possible from a matrix to a vertical line of target holes) in children with dyslexia (Capellini, Coppede, & Valle, 2010; Gooch, Hulme, Nash, & Snowling, 2014) and DLD (DiDonato Brumbach & Goffman, 2014; Finlay & McPhillips, 2013; Flapper & Schoemaker, 2013; Hill, 2001; Jäncke, Siegenthaler, Preis, & Steinmetz, 2007). We are aware of two studies investigating the associations between motor skills at infancy and later speech/language disorders in the same group of children. Viholainen, Ahonen, Cantell, Lyytinen, and Lyytinen (2002) did not find a difference between motor development (measured by parent questionnaires about reaching developmental milestones) of infants with and without a family history of dyslexia. However, children with both a family history of dyslexia and slow motor development at infancy showed weaker language skills at 18 months (Viholainen et al., 2002) as well as slower reading at 7 years of age (Viholainen et al., 2006) than infants without a family history of dyslexia or with a family history of dyslexia but with fast motor development. Taken together, there is mixed evidence for motor impairments in individuals who develop speech/language disorders; further studies in larger samples are needed to disentangle these factors.

The research on infants reviewed here also suggests that the three mechanisms outlined above (fine-grained auditory processing, neural entrainment, and sensorimotor development) are related to speech/language development. Research still needs to determine whether hierarchical processing in infants is related to later speech/language disorders. Even though an impairment in a single domain does not seem to have a discrete oneto-one mapping to specific disorders, we believe that these findings are promising for the use of musical rhythm processing as a potential risk factor, in part because it involves each of the three mechanisms (and potentially other processes shared by musical rhythm and language processing, e.g., precision, emotion, repetition, and attention, see Patel, 2011). Therefore, it is possible that musical rhythm has a stronger association with speech/language disorders than the three mechanisms independently.

## 6 | THE ATYPICAL RHYTHM RISK HYPOTHESIS

In light of the evidence reviewed thus far, we propose the Atypical Rhythm Risk Hypothesis, which posits that individuals with atypical rhythm processing are at higher risk for developmental speech/language disorders. We would like to emphasize that we do not assume that infants with impaired musical rhythm processing will definitively develop a speech/language disorder. Rather, we believe that impaired timing skills measured through music rhythm processing can serve as one risk factor in the prediction of speech/language disorders, in combination with other risk factors both known and still to be determined (Smoller et al., 2019). In practical clinical situations, early screening of rhythm processing with nonverbal, musical material might allow for referral to appropriate speech therapy services for additional testing if atypical rhythm is detected. Broad-based screening of rhythm as a risk factor could offer multiple advantages. First, rhythm skills are likely less affected by the language environment, thus eliminating false positives often occurring in the case of bilingual children when language screeners are used. Second, atypical rhythm may be an indicator of risk for several different speech and language disorders, whereas currently available speech/language screenings tend to be geared toward separate disorders (and thus a child screened for speech difficulties may have an undetected language problem). Third, simple computer-based rhythm assessments could be administered to preschoolers and school-aged children by various professionals (teachers, nurses, school counselors, pediatricians) who do not have specialized Speech–Language Pathology expertise, and then a much smaller number of the children showing atypical rhythm could be referred for SLP assessment, thus optimizing the use of resources. Therefore, our Hypothesis could affect clinical practice first in the screening of preschool-aged and school-aged children, and then could be extended to infant screening when more research and reliable rhythm tests will be available for infants. There are multiple existing behavioral paradigms for measuring rhythm abilities in older children and in adults that could potentially be used for screening. For instance, in rhythm discrimination paradigms (e.g., Gordon, 1979; Law & Zentner, 2012), participants are presented with rhythmic excerpts and asked to decide whether they are the same or different. Tasks from the Battery for the Assessment of Auditory Sensorimotor and Timing Abilities (BAASTA; Dalla Bella et al., 2017) such as tapping in synchrony with the beat of music or deciding whether a beat superimposed onto music is aligned with the beat of the music (see also the Beat Alignment Test, Iversen & Patel, 2008, and the child extension in Einarson & Trainor, 2016) could also inspire screening tasks. Future research should aim to develop rhythm tests with high test–retest reliability for even younger children and for infants.

Recent advances in population genetics analysis methods have highlighted the challenges and opportunities in identifying underlying causal biology that can account for clinically co-morbid conditions of complex traits, such as ADHD, depression, and many other psychiatric disorders (Demontis et al., 2019; Smoller et al., 2019; Tylee et al., 2018). Language and music phenotypes are complex traits, meaning they do not follow a Mendelian pattern of inheritance (Ruiz-Narváez, 2011). The heritability of complex traits is polygenic, that is, involving common genetic variants widely distributed across the genome (Wray et al., 2014), and very large sample sizes are needed to investigate the genetic basis of these traits (Deriziotis & Fisher, 2017; Niarchou et al., 2019). Cross-trait genetic correlation approaches in particular (see Bulik-Sullivan et al., 2015; Turley et al., 2018; Yang, Lee, Goddard, & Visscher, 2011) have revealed a surprising amount of shared underlying genetic architecture (*pleiotropy*; Solovieff, Cotsapas, Lee, Purcell, & Smoller, 2013) across a spectrum of neurodevelopmental and other health disorders (Anttila et al., 2018; Okbay et al., 2016; Watanabe et al., 2005). Recent findings of pleiotropy between ADHD and literacy development (Gialluisi, Andlauer, Mirza-Schreiber, et al., 2019; Verhoef et al., 2019) align with epidemiological evidence of comorbidity (Mueller & Tomblin, 2012), and pave the way for testing the presence and function of underlying shared deficits, such as atypical rhythm processing. The likelihood and feasibility of examining pleiotropy between rhythm and speech/language traits is demonstrated by recent reports of genetic correlations between rhythm and other cognitive and motor traits (i.e., processing speed and grip strength; Niarchou et al., 2019).

Family-based studies have shown that musical rhythm skills are moderately heritable (50%; Ullén, Mosing, Holm, Eriksson, & Madison, 2014), and our ongoing work with genome-wide approaches in a large sample now point to highly polygenic genetic architecture of musical rhythm (Niarchou et al., 2019). While the heritability of musicality traits is a relatively recent area of inquiry (see Gingras, Honing, Peretz, Trainor, & Fisher, 2015, for a review), more is known about the heritability of speech and language abilities. Family history of speech/language disorders has been identified as one of the strongest risk factors for speech/language disorders in offspring. In particular, the literature converges to show moderate to high heritability of speech and language abilities and in particular, of speech and language disorders (dyslexia: Friend, DeFries, & Olson, 2008; Harlaar, Spinath, Dale, & Plomin, 2005; Kirkpatrick, Legrand, Iacono, & McGue, 2011; DLD: Bishop & Hayiou-Thomas, 2008; Conti-Ramsden, Falcaro, Simkin, & Pickles, 2007; Hayiou-Thomas, 2008; stuttering: Dworzynski, Remington, Rijsdijk, Howell, & Plomin, 2007; van Beijsterveldt, Felsenfeld, & Boomsma, 2010; see Deriziotis & Fisher, 2017, for a review). Importantly, different developmental speech/language disorders as well as speech/language disorders and ADHD tend to co-occur in families (e.g., Carroll & Myers, 2010; Flax et al., 2003; Kovac et al., 2001; Lahey & Edwards, 1995; Mueller & Tomblin, 2012).

**FIGURE 4:**
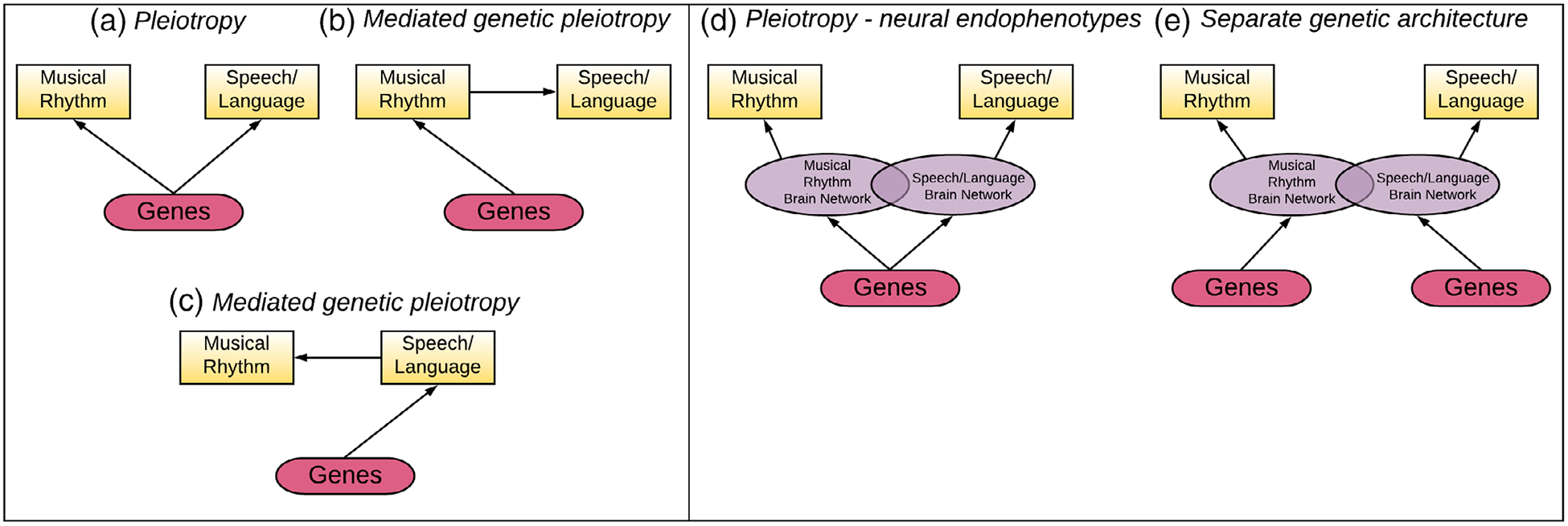
Pleiotropy scenarios for shared versus separate genetic architecture of rhythm and speech/language. The Atypical Rhythm Risk hypothesis predicts that associations between rhythm and speech/language are (a) in part driven by genetic pleiotropy, such that a common set of causal genes affects both phenotypes directly, or (b) mediated genetic pleiotropy, such that genes directly affect rhythm phenotypes, and those phenotypes in turn affect individual differences in acquisition of speech/language during development, or (c) genes directly affect speech/language phenotypes, and those phenotypes affect individual differences in rhythm development. These models should be tested against the null hypothesis of separate genetic architecture. Moreover, a key to understanding the dynamics between genes, brain and behavior will be to test mediating neural endophenotypes linked to (d) shared or (e) separate genetic architecture

As reviewed in earlier sections, there is also converging evidence across approaches and populations for phenotypic correlations between rhythm and speech/language development. Given that phenotypic correlations are generally shown to be driven by some underlying genetic correlation or pleiotropy (Sodini, Kemper, Wray, & Trzaskowski, 2018), it is entirely possible that rhythm and speech/language development share some of their genetic architecture and are mediated through some degree of shared neural architecture. Genetically driven relationships between musical rhythm and speech/language phenotypes might be driven by *pleiotropy*, such that a common set of causal genes affects both phenotypes directly (Figure 4a), or *mediated genetic pleiotropy* (Figure 4b, c). In the case of biological pleiotropy, the same set of genes would affect the development of cortical and subcortical structures underlying musical rhythm processing and speech/language processing. Mediated genetic pleiotropy could occur in both directions: Genes might directly affect rhythm phenotypes, and then those phenotypes affect individual differences in acquisition of speech/language during development (i.e., via enhanced rhythm skills; Figure 4b), or genes could affect speech and language development, which affects the development of musical rhythm skills (Figure 4c); however, we believe this latter case is unlikely given the evidence from training studies reviewed in this article, and the more precise timing necessary for music rhythm processing (see Patel, 2011; Tierney & Kraus, 2014). Future research should investigate the genetic architecture of phenotypic associations between musical rhythm and speech/language processing.

It will also be important to investigate neural endophenotypes (corresponding to overlapping brain networks/mechanisms recruited by both music and speech), that could mediate the relationship between genes, brain, and behavior: A common set of genes may give rise to endophenotypic variation in the brain, that then in turn affects individual variation in both rhythm and language phenotypes (Figure 4d). It is also possible that individual variation in rhythm and language is driven by separate sets of genes, and that phenotypic correlations arise solely due to overlapping brain networks (separate genetic architecture, shown in Figure 4e). Statistical testing of these models will be necessary to disentangle the direction of causation for reported links between atypical rhythm and disordered speech/language acquisition.

The exact mechanisms by which phenotypic relationships are driven are also yet to be understood. If mediated pleiotropy underlies the phenotypic associations between musical rhythm and speech/language processing, it has to be determined what are the exact mechanisms driving the relationship and in what direction. For example, it has been suggested that sound envelope processing as well as synchronization and entrainment to the pulse are shared between music and speech rhythm (SEP hypothesis; Fujii & Wan, 2014), and that precise auditory timing via entrainment to music rhythm can have a positive influence on language processing (PATH hypothesis, Tierney & Kraus, 2014; see also Section 2). One possible scenario (Figure 4b) explaining this relationship would be that allelic variation in genes associated with typical (vs. atypical) rhythm (Niarchou et al., 2019) is involved in the development and maintenance of certain auditory-motor pathways in the brain, that are recruited during rhythmic synchronization and auditory timing, thus enhancing sensitivity to linguistic features of the speech signal and bolstering acquisition of grammar and phonology. In parallel, allelic variation associated with atypical rhythm could result in less-than-optimal development of auditory-motor pathways (measurable via poorer rhythm task performance), resulting in reduced sensitivity to phonological and grammatical information in the speech signal, and increasing the probability of dyslexia or developmental language disorder.

When new studies are deployed to test the Atypical Rhythm Risk Hypothesis, it will also be important to incorporate other risk factors for speech/language disorders. Among the several other risk factors that have been investigated for speech/language disorders (see Mascheretti, Andreola, Scaini, & Sulpizio, 2018), maternal education (Ozernov-Palchik & Gaab, 2016; Sun et al., 2013; Zhao, Zhang, Chen, Zhou, & Zuo, 2016) and, even more so, home literacy environment, seem to be the most important for development dyslexia (Dilnot, Hamilton, Maughan, & Snowling, 2017; Sénéchal & LeFevre, 2002; Storch & Whitehurst, 2001; Sun et al., 2013; Torppa, Eklund, van Bergen, & Lyytinen, 2015; Torppa, Poikkeus, Laakso, Eklund, & Lyytinen, 2006; van Bergen, van der Leij, & de Jong, 2014; Zhao et al., 2016). Preterm birth and birth weight are also found to be risk factors for later language development (Dilnot et al., 2017; Liu et al., 2016; Samuelsson et al., 2006). In DLD, low maternal education level, low 5-min Apgar score, being a male and not being a first child were consistently found to be risk factors according to a meta-analysis (Rudolph, 2017). In stuttering, preterm birth or harmful events before or at birth were proposed as risk factors (Ajdacic-Gross et al., 2010; Stromswold, 2006); less clear is the role of socioeconomic status for this disorder (Yairi & Ambrose, 2013). However, there is strong evidence that all of the risk factors listed in this paragraph arise from gene–environment interactions, and thus it is difficult to dissociate them from other genetic risk factors for speech/language disorders without carefully designed genetic models. Unfortunately, very large-scale population cohort studies such as UK Biobank have generally not included speech/language or musical variables in their massive data collection efforts to date, although genome-wide summary statistics on educational attainment, SES, and preterm birth are now widely available and could be incorporated into novel studies outlined here.

Although we believe that the Atypical Rhythm Risk Hypothesis is a promising view which could facilitate the identification of speech/language disorders, some questions might arise for the reader. One could ask whether associations between musical rhythm and speech/language processing may be explained by other shared processes, such as intelligence, working memory or other general cognitive functions. Although these processes are definitely involved in both domains, it does not undermine the Atypical Rhythm Risk Hypothesis. First, associations between musical rhythm and speech/language processing were found to be associated after controlling for the variance in general cognitive measures (e.g., Gordon, Shivers, et al., 2015). Second, if musical rhythm at infancy or early childhood proves to be a sufficient risk factor for speech/language disorders, for practical purposes it is irrelevant what the underlying shared processes are. One could also ask why we propose that a non-linguistic impairment might contribute to speech/language disorders. Even though this contradicts some prevalent domain-specific views about speech/language disorders, it is in line with the contemporary view of speech/language disorders stating that not only linguistic processes are impaired in these populations (e.g., Hill, 2001; Ullman & Pierpont, 2005). The reader could also wonder how rhythm tests would be integrated in clinical practice; we intentionally aim to be cautious and only focus in the current paper on research that must first be conducted before the potential integration of rhythm screeners into clinical practice. If research findings result in support of rhythm tests as a screener, the details of such implementation into clinical practice should be determined by experts from developmental research, speech–language pathology, and policy-makers depending on local and national systems in place.

## 7 | CONCLUSION

Inefficient identification of speech/language problems has academic, social, and economic consequences both for the affected individuals, their families, and society (e.g., Conti-Ramsden et al., 2018; Snow, 2019). In the current paper, we reviewed evidence motivating the Atypical Rhythm Risk Hypothesis, which posits that individuals with atypical rhythm are at higher risk for developmental speech/language disorders. We reviewed different lines of research suggesting (a) shared underlying processes for musical rhythm and speech/language processing, (b) associations between musical rhythm and speech/language processing in typically developing populations and impaired musical rhythm processing in children with developmental disorders affecting speech/language skills, and (c) individual differences in mechanisms underlying rhythm processing in infants, which were associated with later speech/language development.

The Atypical Rhythm Risk Hypothesis and its theoretical framework presented here allow us to generate a series of predictions (presented in Box 2) about co-morbidities between rhythm and speech/language disorders and the shared underlying biology from genes to brain to behavior. For instance, one prediction of our theory is that if a screener for atypical rhythm is administered to a large population, individuals with rhythm deficits would show a higher prevalence of speech/language disorders. Associations between rhythm-related processes at infancy and language development reported by Kalashnikova et al. (2019) provide initial supporting evidence for this hypothesis. Furthermore, there is already some evidence for statistically increased prevalence rates of atypical rhythm in association with developmental disorders (Peretz & Vuvan, 2017) from a study of over 16,000 individuals, which identified 2.7% of their sample as “time-based amusics” and an additional 3.4% of the sample with general amusia/uncategorized deficits that included poor rhythm performance. In particular, the time-based amusia group had higher prevalence of dyslexia, speech disorders, and attentional disorders than controls, consistent with the framing of atypical rhythm as a risk factor across these disorders. Although the contribution of pitch-based amusia and deficits in other aspects of music perception to atypical speech/language development (i.e., Couvignou, Peretz, & Ramus, 2019) is beyond the scope of the current review, other elements of musicality should certainly be considered in the broader context of an influence of rhythm, melody, timbre, or harmony on speech and language development (see Brandt, Gebrian, & Slevc, 2012, for a model of how “musical hearing” may scaffold language acquisition).

The Atypical Rhythm Risk Hypothesis is in line with the transdiagnostic approach (Mareva & Holmes, 2019) emphasizing the need for large-scale epidemiological studies (e.g., Raghavan et al., 2018). This work needs to incorporate various known and to-be-determined risk factors into prediction models and disentangle gene–environment interactions, intermediary neural endophenotypes, and underlying biological mechanisms. Once genome-wide data for rhythm and language phenotypes from large enough samples are available, recently developed methods such as two-sample Mendelian Randomization (Zhu et al., 2018) may be used to begin to identify the hypothesized causal influence of rhythm on speech/language development (even when measured in separate samples) and to model other contributing variables. With new population-based efforts to assess individual differences in rhythm and speech/language abilities in tens and hundreds of thousands of participants (i.e., Niarchou et al., 2019, and ongoing work by the GenLang consortium), exploration of the hypothesized shared genetic architecture among these traits and other risk factors (e.g., Watanabe et al., 2005) is on the near horizon.

If a significant body of experimental evidence is found in favor of the hypothesized association between atypical rhythm and speech/language disorders, we can envision new risk factor models that incorporate atypical rhythm processing. Measuring rhythm processing could serve as a simple, easy-to-administer prescreening test that can be conducted with young infants and children and even in parents to identify familial risk of atypical rhythm. These screening efforts could be used as a tool to increase referrals to appropriate speech/language pathology services with the end goal of closing the gap in the identification and increasing access to early intervention to maximize long-term impact.

## Figures and Tables

**TABLE 1:** Summary of current literature investigating rhythm in children with atypical speech–language development

		Age	Task	Evidence for atypical rhythm?
Dyslexia	Colling, Noble, and Goswami (2017)	9–10 years	Beat perception Tapping task	Yes
	Cutini, Szucs, Mead, Huss, and Goswami (2016)	12 years	Neural entrainment to amplitude-modulated noise	Yes (2 Hz)
	Frey, Francois, Chobert, Besson, and Ziegler (2019)	10 years	Neural processing of speech sounds in silence, noise, and envelope conditions	Yes
	Goswami et al. (2002)	11 years	Beat detection in amplitude-modulated sounds	Yes
	Goswami, Gerson, and Astruc (2010)	7–13 years	Amplitude envelope onset (rise time) discrimination	Yes
	Goswami, Huss, Mead, Fosker, and Verney (2013)	8–14 years	Beat perception	Yes
	Goswami et al. (2013)	9 years	Syllable stress discrimination	Yes
	Goswami et al. (2016)		Discrimination of amplitude rise time	Yes
			Temporal modulations of nursery rhymes	No but impaired acoustic learning during the experiment from low-pass filtered targets
	Hämäläinen, Rupp, Soltész, Szücs, and Goswami (2012)	19–29 years	Amplitude-modulated white noise	Yes at 2 Hz
	Huss, Verney, Fosker, Mead, and Goswami (2011)	8–13 years	Amplitude envelope rise time perception	Yes
	Lee, Sie, Chen, and Cheng (2015)	9–12 years	Rhythmic imitation	Yes
	Leong and Goswami (2014)	<40 years, mean: 22 years	Rhythmic detection to identify amplitude-modulated nursery rhyme sentences	Yes
	Leong, Hämäläinen, Soltész, and Goswami (2011)	17–41 years	Amplitude envelope onset (rise time) perception and syllable stress detection	Yes
	Lizarazu et al. (2015)	Children: 8–14 years; adults: 17–44 years	Auditory neural synchronization	Yes
	Molinaro, Lizarazu, Lallier, Bourguignon, and Carreiras (2016)	Children: 8–14 years; adults: 22–37 years	Neural synchronization to spoken sentences (MEG)	Yes
	Muneaux, Ziegler, True, Thomson, and Goswami (2004)	11 years	Beat perception (slope)	Yes
	Overy (2000)	6–7 years	Rhythm discrimination Tempo discrimination Meter reproduction	Yes, especially in meter reproduction
	Overy, Nicolson, Fawcett, and Clarke (2003)	7–11 years	Tests of timing skills (rhythm copying, rhythm discrimination, song rhythm, tempo copying, tempo discrimination, song beat)	Yes
	Pasquini, Corriveau, and Goswami (2007)	19–27 years	Rise time perception and temporal order judgment	Yes
	Persici et al. (2019)	9–11 years	Tapping	Yes
	Power, Colling, Mead, Barnes, and Goswami (2016)	12–14 years	Neural entrainment to speech syllables	Yes
	Soltész, Szűcs, Leong, White, and Goswami (2013)	Mean: 25.8 years	Neural entrainment to tones presented at 2 or 1.5 Hz	Yes
	Surányi et al. (2009)	8–9 years	Amplitude envelope rise time discrimination	Yes
	Thomson, Fryer, Maltby, and Goswami (2006)	18–31 years	Basic auditory processing tasks (rise time, duration, and intensity discrimination)	Yes
			Tempo discrimination	No
			Tapping (unimanual and bimanual)	Yes but only in the inter-tap-interval variability
	Thomson and Goswami (2008)	10 years	Rhythmic discrimination	No
			Paced and unpaced finger tapping	Yes
	Wang, Huss, Hämäläinen, and Goswami (2012)	9–10 years	Basic auditory processing tasks (rise time, duration, and intensity discrimination)	Yes
	Zuk et al. (2017)	18–36 years	Speech syllable discrimination	Yes
DLD	Bedoin et al. (2016)	9–11 years	Rhythm discrimination	Yes
	Corriveau and Goswami (2009)	7–11 years	Paced and unpaced tapping	Yes in the paced condition
	Corriveau, Pasquini, and Goswami (2007)	7–11 years	Amplitude envelope rise time and sound duration perception	Yes
	Cumming, Wilson, Leong, Colling, and Goswami (2015)	6–12 years	Beat detection Tapping Speech/music task	Yes, especially in tapping
	Goswami et al. (2016)	9 years	Discrimination of amplitude rise time	Yes
			Temporal modulations of nursery rhymes	
	Richards and Goswami (2015)	8–12 years	Stress perception task	Yes
	Richards and Goswami (2019)	6–11 years	Stress pattern disruptions	Yes
	Sabisch, Hahne, Glass, von Suchodoletz, and Friederici (2009)	8–10 years	Syntactic processing with prosody disruptions	Yes
	Sallat and Jentschke (2015)	4–5 years	Rhythmic–melodic perception task	Yes
	Vuolo, Goffman, and Zelaznik (2017)	4–5 years	Tapping and bimanual clapping	Yes, but only in the bimanual clapping task
	Weinert (1992)	5–8 years	Rhythmic discrimination	Yes
	Wells and Peppé (2003)	8 years	Prosody perception	Yes
	Zelaznik and Goffman (2010)	6–8 years	Tapping and drawing to a metronome	Yes (but no in the timing skill in the manual domain)
Stuttering	Chang, Chow, Wieland, and McAuley (2016)	6–11 years	Auditory rhythm discrimination task	Yes
	Falk, Müller, and Dalla Bella (2015)	8–16 years	Finger tapping	Yes
	Olander, Smith, and Zelaznik (2010)	4–6 years	Metronome clapping	Yes
	Toyomura, Fujii, and Kuriki (2011)	18–55 years	Metronome-timed speech	No (yes in the normal speech condition)
	Wieland, McAuley, Dilley, and Chang (2015)	6–11 years	Simple and complex rhythms discrimination	Yes
DCD	Puyjarinet, Begél, Lopez, Dellacherie, and Dalla Bella (2017)	Children: 6–12 years; adults: 19–50 years	Duration and beat perception Tapping	Yes
	Rosenblum and Regev (2013)	7–12 years	Metronome synchronization	Yes
	Trainor, Chang, Cairney, and Li (2018)	6–7 years	Auditory duration and rhythm discrimination Oddball ERP paradigm	Yes
ADHD	Carrer (2015)	6–14 years	Rhythmic discrimination	Yes
	Hove, Gravel, Spencer, and Valera (2017)	20 years	Paced and unpaced finger tapping	Yes (in the standard task, not in the one with time shifts)
	Puyjarinet et al. (2017)	Children: 6–12 years; adults: 19–50 years	Duration and beat perception Tapping	Yes
	Valera et al. (2010)	10 years	Paced and unpaced tapping	Yes—greater within-subject variability
	Zelaznik et al. (2012)	9 years	Spacebar press following a metronome	Yes

Abbreviations: ADHD, attention deficit hyperactivity disorder; DCD, developmental coordination disorder; DLD, developmental language disorder.

## BOX 1 Glossary of terms

### Attention deficit hyperactivity disorder.

Attention deficit hyperactivity disorder involves difficulties with attention, hyperactivity, and impulsivity (Biederman & Faraone, 2005), and is frequently associated with language impairment (e.g., Randell, Somerville-Brown, & Chen, 2018).

### Developmental coordination disorder.

Developmental coordination disorder is characterized by impaired motor abilities, especially related to postural control, motor learning, and sensorimotor coordination that affect quality of life (Zwicker et al., 2009). Difficulties with language processing were also reported for DCD (e.g., Mirabella et al., 2017).

### Developmental dyslexia.

Dyslexia is a neurodevelopmental disorder affecting word decoding, which in turn impacts spelling performance and the development of reading fluency (Snowling, 2013).

### Developmental language disorder (DLD).

DLD is a disorder affecting language comprehension and/or production, with no known biomedical causes such as brain injury, acquired epileptic aphasia, neurodegenerative disease, cerebral palsy, hearing loss, intellectual disability, or autism spectrum disorder. DLD is likely to persist in middle childhood and beyond (Bishop et al., 2017). In accordance with recent recommendations (Bishop et al., 2017) we use the term developmental language disorder for children who were often referred to as children with specific language impairment in previous studies. Bishop et al. (2017) suggest eliminating the latter term because it is misleading to state that the impairment is specific to language. In addition, they suggest using the term *disorder* instead of impairment to convey greater seriousness and importance, and the naming consistent with the names of other developmental disorders (Autism spectrum disorder, developmental coordination disorder, attention deficit hyperactivity disorder) and compatible with the two main diagnostic systems (DSM-5 [American Psychiatric Association, 2013] and ICD-11 [World Health Organization, 2018]).

### Heritability.

Heritability is the proportion of phenotypic variation that is due to genetic variation. Heritability analyses also allow for a comparison of the relative importance of genes and environment to the variation of traits within and across populations (Visscher, Hill, & Wray, 2008).

### Mismatch negativity (MMN).

MMN appears when a regular feature of a sound sequence is infrequently omitted (Kujala, Tervaniemi, & Schröger, 2007; Näätänen, Tervaniemi, Sussman, Paavilainen, & Winkler, 2001).

### Pleiotropy.

Pleiotropy occurs when one genetic locus affects more than one trait (Solovieff et al., 2013).

### Rapid auditory processing.

The ability to process and categorize fast acoustic changes over a millisecond time range (e.g., detecting changes in pitch of tones when they are presented with very short [70 ms] inter-stimulus intervals; Benasich et al., 2006).

### Risk factors for language disorders.

Risk factors are biological or environmental factors that are statistically associated with language disorders, but whose causal relationship to the language problem is unclear or partial (Bishop et al., 2017).

### Stuttering.

Stuttering is a speech disorder characterized by frequent occurrences of repetitions or prolongations of sounds, syllables, or words that disrupt the rhythmic flow of speech (World Health Organization, 2010). Language impairments often co-occur with stuttering (Ntourou, Conture, & Lipsey, 2011).

### Transdiagnostic approach.

The transdiagnostic approach focuses on underlying mechanisms that are relevant across a class of disorders instead of single diagnostic categories in research, diagnosis and treatment of disorders (see Sauer-Zavala et al., 2017).

## BOX 2 Cumulative evidence and predictions of the Atypical Rhythm Risk Hypothesis for future research

Current evidence for the Atypical Rhythm Risk Hypothesis

- **Individual differences in rhythm ability** are associated with speech/language skills as well as task performance in TD children and adults; there is some overlap in neural circuitry.
- **Associations are found among underlying mechanisms of rhythm processing:** fine-grained auditory processing, entrainment of neural oscillatory networks, motor function, and hierarchical processing; all are used in speech perception/production.
- **Rhythm impairments are prevalent in children with atypical speech-language development** (e.g., dyslexia, DLD, stuttering) and in commonly co-morbid motor and attentional disorders.

Predictions of the Atypical Rhythm Risk Hypothesis

- **Individual differences approaches**
  - Individual differences in rhythm sensitivity (measured across the lifespan with various behavioral and physiological methods) will predict significant variance in speech/language skills cross-sectionally and longitudinally. When assessing rhythm in infants, it will be important to discover the youngest age range at which atypical rhythm could predict risk for later speech/language disorders.
- **Rhythm screening approaches**
  - We predict higher prevalence of speech/language disorders and more generally, lower linguistic performance/skills among individuals (children, adults) who show poor performance on rhythm tasks
  - Young children with atypical rhythm sensitivity are more likely to develop speech/language disorders.
  - Rhythm screening tests could eventually be used clinically to refer to Speech/Language Pathology services for more extensive testing.
    - If the rhythm screening approach turns out to be promising, intervention programs, potentially incorporating a rhythm component, could be tested.
- **Family-based predictions**
  - Parents with atypical rhythm sensitivity are more likely to have children with atypical rhythm sensitivity and who develop speech/language disorders.
  - Predictive power could be improved by combining atypical rhythm and other risk factors for speech/language disorders into a “risk score”.
- **Genetic correlations between rhythm and speech/language phenotypes**
  - We predict some degree of shared genetic architecture between rhythm and speech/language development that would account for phenotypic correlations.
  - Shared underlying genetic architecture could affect overlapping neural circuitry as an endophenotype between rhythm and speech/language development.
  - A causal influence of rhythm on language acquisition can be assessed with genetic modelling (See Figure 4), and alternative models can be compared.
  - Gene-environment interactions could also be assessed with twin models or other family-based methods if there is data on speech/language and rhythm phenotypes available within a given (large) cohort.

## Footnotes

1 Note that musical rhythm processing, speech/language functions, and motor impairments were reported in nondevelopmental disorders such as Parkinson’s disease and in acquired brain injuries (e.g., Ariatti, Benuzzi, & Nichelli, 2008; Grahn & Brett, 2009; Kotz & Gunter, 2015; Smith & Caplan, 2018). The investigation of the relationship between these impairments has a theoretical importance as well as clinical relevance. This article and the Atypical Rhythm Risk Hypothesis, however, focus on developmental disorders, and the extension to nondevelopmental disorders is beyond the scope of the paper.
